# Development and Characterization of Semi-Refined Iota Carrageenan/SiO_2_-ZnO Bionanocomposite Film with the Addition of Cassava Starch for Application on Minced Chicken Meat Packaging

**DOI:** 10.3390/foods10112776

**Published:** 2021-11-11

**Authors:** Danar Praseptiangga, Dea Widyaastuti, Camellia Panatarani, I Made Joni

**Affiliations:** 1 Department of Food Science and Technology, Faculty of Agriculture, Universitas Sebelas Maret (UNS), Jl Ir. Sutami 36 A, Kentingan, Jebres, Surakarta 57126, Central Java, Indonesia; deawidyaastuti@student.uns.ac.id; 2 Functional Nano Powder University Center of Excellence, Universitas Padjdajaran, Jl Raya Bandung-Sumedang KM 21, Jatinangor, Sumedang 45363, West Java, Indonesia; c.panatarani@phys.unpad.ac.id (C.P.); imadejoni@phys.unpad.ac.id (I.M.J.); 3 Department of Physics, Faculty of Mathematics and Natural Sciences, Universitas Padjadjaran, Jl Raya Bandung-Sumedang KM 21, Jatinangor, Sumedang 45363, West Java, Indonesia

**Keywords:** carrageenan, starch, nanocomposite film, minced chicken meat, food packaging

## Abstract

In the current study, film based on semi-refined ι-carrageenan/cassava starch (SRiC/CS) incorporated with SiO_2_-ZnO nanoparticles was fabricated and characterized to deal with serious environmental problems resulting from plastic packaging materials. This study aimed to evaluate film properties with the variation of SRiC/CS proportions of bionanocomposite films for application to minced chicken meat packaging. Increasing CS portion contributed to increased transparency, reduced surface roughness, and decreased mechanical properties of films. The variable significantly (*p* < 0.05) increased the water vapor permeability (WVP) and reduced the water solubility of films. The incorporation of the nanoparticles significantly (*p* < 0.05) increased UV screening, decreased WVP, and enhanced the antimicrobial activity of films. Furthermore, the substitution of 0.5 wt% (weight percentage) CS provided the best film characteristics. Based on the color and the total volatile base nitrogen (TVBN) results, SRiC film incorporated with the nanoparticles preserved minced chicken quality up to six days. Thus, the developed films are desirable for biodegradable food packaging.

## 1. Introduction

Plastic is a multi-purpose and petrochemical-based material, which is non-degradable and causes environmental problems. Annual global plastic production reached 359 million tons in 2018, mainly plastic-based packaging, and keeps rising [1]. Consequently, the accumulation of plastic waste is potentially transformed into microplastics and contaminates water, which is risky for humans and living organisms [2]. Therefore, the investigation of biodegradable plastic/film is attractive for many researchers.

Carrageenan is a linear sulfated polysaccharide that consists of a repeating disaccharide unit with 3,6-anhydrous-α-galactopyranose, which is a prospective material for biodegradable film [3]. The ester sulfate group is associated with the gelling capability of ι-carrageenan and κ-carrageenan, which does not exist in λ-carrageenan [4]. A low concentration of ι-carrageenan could form gels in aqueous media, which are transparent, thermoreversible, and have various textures from highly elastic to cohesive [5]. Semi-refined carrageenan-based film has not been widely studied due to impurities that affect the optical and mechanical properties of the film. The shorter processing steps of semi-refined carrageenan than refined carrageenan still leave 20 to 30% of cellulosic and other residual plant debris constituents, which affect the optical and mechanical properties of the film. However, the film is more stretchable than refined carrageenan film [6]. Based on previous works, the development of semi-refined ι- or κ-carrageenan film incorporated with SiO_2_ or ZnO nanoparticles requires an alternative approach and food packaging application [7,8,9].

Previous studies revealed that incorporation of SiO_2_-ZnO nanoparticles successfully enhanced semi-refined ι-carrageenan (SRiC) film properties [10] and improved the hydrophobicity and antimicrobial activity of PVA-chitosan film [11]. Moreover, a mixture of Ag, ZnO, and CuO nanoparticles reinforced the mechanical and antimicrobial properties of the active starch film [12]. However, biodegradable plastic has been competing against lower prices of conventional plastic [13]. Thus, cassava starch (CS) was combined with SRiC to produce affordable film packaging due to its availability, biodegradability, and film-forming capability, which results in transparency, high impermeability to oxygen, and stretchable film [14]. Furthermore, the addition of CS to chicken skin gelatin-based film promoted film properties [15].

OECD/FAO (2017) estimated that global poultry production would increase from 117 Mt to 132 Mt in 2026, mainly through chicken production [16]. Moreover, consumer preference for minced chicken is correlated with healthy, low-fat, and minimally processed food [17]. However, minced chicken in the retail market tends to putrefy. Thus, refrigeration and packaging are necessary to maintain meat quality and safety [18]. Therefore, this study aimed to analyze the film characteristics due to the effect of the addition of CS on SRiC incorporated with SiO_2_-ZnO nanoparticles and then evaluated the application of fabricated film to minced chicken.

## 2. Materials and Methods

### 2.1. Materials

Semi-refined ι-carrageenan (SRiC) and cassava starch (CS) were purchased from Galic Artabahari, Co., Ltd. (Bekasi, Indonesia) and Budi Starch & Sweetener, Co., Ltd. (Lampung, Indonesia), respectively. SiO_2_ and ZnO nanoparticle powder with a particle size of about ±50 nm and 100–200 nm, respectively, were obtained from JP Cipta Nanotech Indonesia Ltd. (Bandung, Indonesia). Sodium dodecyl sulfate (SDS) and glycerol were purchased from Brataco, Co., Ltd. (Bandung, Indonesia). *E. coli* FNCC 0091 and *S. aureus* FNCC 0047 were obtained from the Food Nutrition and Culture Collection (FNCC), Universitas Gadjah Mada (Yogyakarta, Indonesia). NaCl, nutrient agar (NA), nutrient broth (NB), and plate count agar (PCA) were purchased from Merck, Co. (Darmstadt, Germany). Distilled water was used for film preparation. Other chemicals and reagents were of analytical grade and used as received.

### 2.2. Preparation of Nanoparticle Suspension

Briefly, 0.1 g SiO_2_ and 0.3 g ZnO nanoparticle powder was dispersed in distilled water and stirred for 1 h as prepared in a previous study [10]. Then, SDS (10 wt% nanoparticles) was added under stirring for 1 h; the total weight of the suspension was 150 g. Subsequently, this suspension was sonicated for 30 min under cold conditions and bead milling for 120 min to provide well-dispersed and stable nanoparticle suspension. Furthermore, the prepared nanoparticle suspension for fabricating bionanocomposite films was about 15 g and was dispersed in distilled water (to obtain 100 g total film suspension) and then sonicated for 30 min before being used [19].

### 2.3. Preparation of Bionanocomposite Films

Bionanocomposite films consist of SRiC-based film as control film (F0) and SRiC film incorporated with SiO_2_-ZnO nanoparticles (F1); then, following the ratio of substitution of SRiC (2 wt% of total film suspension) with CS, i.e., 1.5 wt%: 0.5 wt% (F2), 1.0 wt%: 1.0 wt% (F3), 0.5 wt%: 1.5 wt% (F4), incorporated with SiO_2_-ZnO nanoparticles. Firstly, SRiC and CS powder were dry-mixed according to the film formula (F2 to F4). Based on the preliminary study, the mixture was dissolved in the nanoparticle suspension and heated at 80 °C under constant stirring for 20 min. Subsequently, the mixture was cooled to 70 °C and glycerol (1 wt%) was added, and the temperature was maintained for 10 min. Afterward, the film-forming suspension was cooled to 45 °C and cast onto a plastic plate (24 × 16 × 2 cm^3^). Afterward, it was cooled for 15 min and dried in a drying oven at 50 °C for 3 h. Finally, it was peeled off and stored in a dry cabinet (27 °C, 57% RH) until being tested. The control film (F0) and F1 were fabricated by dissolving 2 g of SRiC in 100 g distilled water and nanoparticle suspension, respectively. The film suspension was heated to 70 °C, and glycerol (1 wt%) was added, followed by the same procedure without being heated at 80 °C for 20 min [8,10].

### 2.4. Characterization of Bionanocomposite Films

#### 2.4.1. Particle Size Distribution and Zeta Potential

The particle size distribution and zeta potential of SiO_2_-ZnO nanoparticle suspensions were measured using a nanoparticle size analyzer (HORIBA nanoparticle SZ-100, HORIBA, Kyoto, Japan) with the dynamic light scattering (DLS) method and electrophoretic light scattering method, respectively.

#### 2.4.2. FTIR Analysis

The FTIR spectra of developed films were obtained by Attenuated Total Reflectance-Fourier Transform Infrared spectrophotometer (ATR-FTIR Thermo Nicolet iS5, Thermo Fisher Scientific, Waltham, MA, USA) analysis in the range of 4000–400 cm^−1^ with OMNIC Spectra software operated at a resolution of 4 cm^−1^.

#### 2.4.3. Thickness

The thickness of each film was assessed at ten randomly selected points using a digital micrometer (KRISBOW KW06-86, KRISBOW, Jakarta, Indonesia) with 0.001 mm of precision, and the average values were recorded. The film thickness was used for water vapor permeability and tensile strength calculations.

#### 2.4.4. Optical Properties

The optical properties of fabricated films were evaluated using an ultraviolet–visible spectrometer (Shimadzu UV1800, Shimadzu, Tokyo, Japan) in the range of 200–900 nm. The film sample (3 × 1 cm^2^) was pasted on the clear side of the cuvette wall, and air was used as the reference. UV screening and transparency of the films were measured as the transmittance (%) at a wavelength of 280 nm (T_280_) and 660 nm (T_660_), respectively.

#### 2.4.5. Film Morphology Analysis

The morphological surface of developed films was analyzed using a scanning electron microscope (SEM HITACHI model SU3500, HITACHI, Tokyo, Japan) and an atomic force microscope (AFM). SEM analysis was conducted at an acceleration voltage of 10 kV under vacuum conditions. Before the analysis, the samples were coated with a thin layer of gold to improve SEM imaging as the film sample is a non-conductive material.

#### 2.4.6. Contact Angle and Critical Surface Tension

The water contact angle of fabricated films was measured to determine the hydrophobic/hydrophilic of the film surface based on the sessile drop technique, according to Lamour et al. [20]. The deionized water (2 µL) drop on the film surface was captured with a digital camera, and the image was analyzed using ImageJ software to determine the contact angle (θ). Furthermore, the contact angle measurement of three different liquids (deionized water, formamide, and n-hexadecane) was applied to determine the critical surface tension of the film surface. The critical surface tension (γ_c_) of the samples was calculated using the linear regression of surface tension (γ) of each liquid as the *x*-axis and the *cos* value of the contact angle (θ) as the *y*-axis (*cos* θ = 1). Based on Fox-Zisman approximation, when *cos* θ = 1, then γ = γ_c_. 

#### 2.4.7. Water Vapor Permeability (WVP)

The WVP of developed films was gravimetrically determined using the dry cup method based on ASTM E96/95 (2005) with modifications [10,21]. The test cup was filled with 3 g anhydrous CaCl_2_, covered with the tested films, sealed with melted paraffin, and placed in a humidity chamber (25 °C, 90% RH). The change in the test cup weight was measured every hour for 7 h and recorded as a function of time. The WVP was calculated according to Equation (1):

(1) $$\mathrm{WVP}=\frac{\mathrm{WVTR}\;x}{P\;({\mathrm{RH}}_{1}-{\mathrm{RH}}_{2})}$$

where WVP is expressed as 10^−10^ g/s m Pa, WVTR (Water Vapor Transmission Rate) is the slope (g/h) divided by the transfer area (m^2^), x is the tested film thickness, P is the saturated water vapor pressure at 25 °C, RH_1_, and RH_2_ are the RH inside the humidity chamber and the test cup, respectively.

#### 2.4.8. Water Solubility

The water solubility of developed films was determined according to Basiak et al. with modifications [22]. The tested films (3 × 3 cm^2^) were dried (100 °C, 6 h) and weighed as the initial dry weight of the films (w_i_). The film samples were individually immersed in 50 mL of distilled water and continuously stirred at 25 °C for 12 h. The insoluble matter of the films was filtered using filter paper and dried (100 °C, 6 h) to determine the final dry weight of the films (w_f_). Then, the film solubility in water was calculated using Equation (2):

(2) $$\mathrm{Solubility}\;(\%)=(\frac{w_{i}-w_{f}}{w_{i}})\times 100$$

#### 2.4.9. Mechanical Properties

The mechanical properties of developed films, such as tensile strength (TS) and elongation at break (EAB), were measured using a tensile testing machine (Universal Testing Machine Zwick type 0.5, Zwick Roell Engineering Corporation, Kennesaw, USA). The film sample was cut (50 × 5 mm^2^) and tested under a pre-load value of 2 N/mm^2^, a test speed of 10 mm/minute, and initial grip of 50 mm. The test result was recorded by computer, and the stress-strain curve as the result with the average film thickness was applied to determine TS and EAB.

#### 2.4.10. Thermal Stability

The thermal stability of developed films was evaluated by the Differential Thermal Analysis-Thermo gravimetric Analysis (DTA-TGA) method using a thermal analyzer (TA instruments) with modifications. The film samples (2 mg) were heated from room temperature to 500 °C. The heating rate and nitrogen flow were maintained at 10 °C/min and 25 mL/min, respectively. Then, the derivative form of TGA (DTGA) was calculated using the forward finite difference method based on TGA values as Equation (3):

(3) $$\mathrm{DTGA}=\frac{\left(w_{t+\Delta t}-w_{t}\right)}{\Delta t}$$

where w_t+Δt_ and w_t_ are the residual weight of the film sample at time t + Δt and t, respectively, and Δt is the time interval for reading the residual weight of the film sample [23].

#### 2.4.11. Antimicrobial Activity

The antimicrobial activity of developed films was evaluated using the disc diffusion agar method, which was described by Chollakup et al. with modifications [24]. Food-pathogenic bacteria, such as *Escherichia coli* and *Staphylococcus aureus*, were used and cultured in the sterile nutrient broth (35 °C, 24 h) before the analysis. The bacterial culture was transferred to the sterile nutrient broth until the concentration was approximately 1–2 × 10^−8^ CFU/mL (0.5 McFarland) and seeded on the nutrient agar using a sterile glasss rod spreader. Then, the film sample (5 mm diameter) was placed on the agar plates and incubated at 35 °C for 24 h. The antimicrobial activity of the film was determined by measuring the diameter of the inhibition zone (clear zone around the sample) with a caliper.

#### 2.4.12. Biodegradability

The biodegradability of developed films was assessed by the soil burial test method, which was described by Maran et al. with modifications [25]. The film samples (3 × 3 mm^2^) were buried at a depth of 10 cm from the soil surface in a bucket and weighed every seven days for 28 days. The bucket containing the sample was covered to maintain its humidity and incubated at room temperature in the laboratory. The degradation rate was determined by measuring the weight loss of the film sample with Equation (4):

(4) $$\mathrm{Weight}\;\mathrm{loss}\;(\%)=(\frac{w_{a}-w_{b}}{w_{a}})\times 100$$

where w_a_ and w_b_ are the film weight before and after degradation.

### 2.5. Application of Bionanocomposite Film for Minced Chicken Meat Packaging

The minced chicken meat was purchased from the local supermarket and transported to the laboratory in a cool box. The application was conducted with a test cup, filled with 50 g minced meat, and covered by a selected film under aseptic conditions. Subsequently, the sample was stored at refrigeration temperature for 12 days and tested every three days. The microbiological quality of meat samples was evaluated using the total plate count (TPC) method [26]. The pH of the samples was measured by using a digital pH meter [27]. The water-holding capacity (WHC) was analyzed by using a low-speed centrifugation method [28]. Sample weight loss was investigated according to Campanone et al. [29]. Meat color was analyzed by using a chromameter (Konica Minolta CR-400) with the CIE-L*a*b* system [30]. Thiobarbituric acid analysis (TBA) was conducted by using the distillation method [31]. Then, total volatile base nitrogen (TVBN) was analyzed according to Senapati and Sahu [32].

### 2.6. Statistical Analysis

The film characterization tests were carried out in triplicate. Then, the application of bionanocomposite film analysis was carried out in duplicate, and two films per sample were investigated. The results were presented as the mean ± SD (standard deviation). Then, the statistical analysis of data was performed by one-way analysis of variance (ANOVA) using the IBM SPSS Statistics 23.0 program. The difference between mean values of the result was compared using Duncan’s multiple range tests (DMRT) at the 0.05 level of significance.

## 3. Results and Discussion

### 3.1. Characterization of Bionanocomposite Films

#### 3.1.1. Particle Size Distribution

The SiO_2_-ZnO nanoparticle suspension was successfully dispersed by bead milling and has been reported previously. Bead milling decreased the particle size distribution (from 2455.6 nm to 263.3 nm) and dispersion stability of the suspension with an increase in the zeta potential value (from −73.1 mV to −26.5 mV) [19].

#### 3.1.2. FTIR Analysis

**Figure 1:**
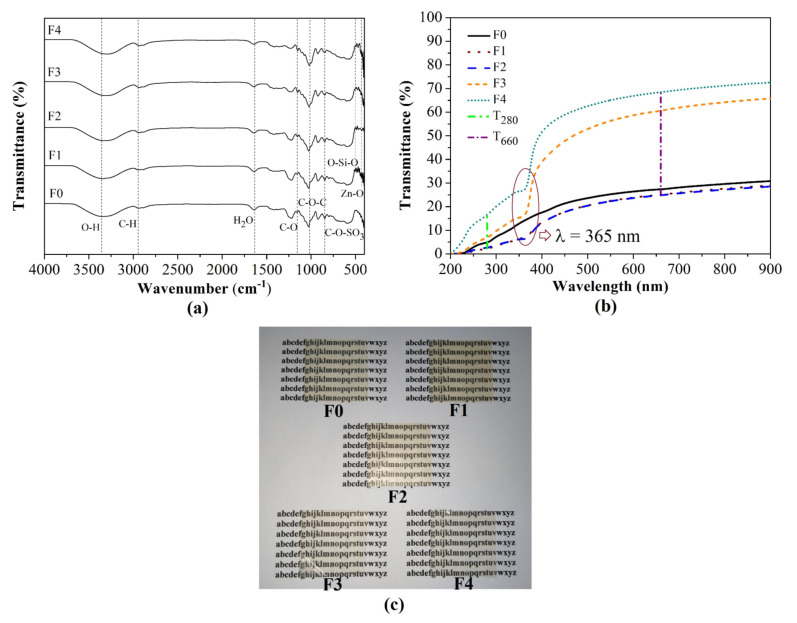
FTIR spectra (**a**), UV light transmittance (**b**), and appearance (**c**) of bionanocomposite and control films. F0: SRiC film (control film), F1: SRiC film with SiO_2_-ZnO nanoparticles, F2: SRiC/CS (1.5 wt%: 0.5 wt%) with SiO_2_-ZnO nanoparticles, F3: SRiC/CS (1.0 wt%: 1.0 wt%) with SiO_2_-ZnO nanoparticles, F4: SRiC/CS (0.5 wt%: 1.5 wt%) with SiO_2_-ZnO nanoparticles, T_280_: transmittance value at 280 nm, and T_660_: transmittance value at 660 nm.

FTIR analysis of the SiO_2_-ZnO nanoparticle suspension was published previously [33]. The FTIR spectra of fabricated films are shown in Figure 1a. The broad band around 3380 cm^−1^ is attributed to the –OH stretching band [34]. The bands around 430 to 520 cm^−1^ and 473 cm^−1^ indicate Zn-O and O-Si-O bands, respectively [34,35]. The bands at 1641 cm^−1^ and 844 cm^−1^ are ascribed to H_2_O and C-O-SO_3_ groups, respectively [36]. The addition of CS shifted the –OH groups to lower wavenumber (from 3344.11 to 3297.30 cm^−1^), indicating that intermolecular interaction between polymers and nanoparticles became weaker [34]. The H_2_O and C-O-SO_3_ groups shifted to a higher wavenumber (from 1642.49 to 1645.40 cm^−1^ and from 846.05 to 847.72 cm^−1^, respectively), indicating the higher hygroscopicity and lower gelling ability of the films [36]. Thus, polymer and nanoparticles physically interacted, without forming a new functional group.

#### 3.1.3. Appearance, Thickness, and Optical Properties of Bionanocomposite Films

Figure 1c illustrates that the F0, F1, and F2 films were yellowish, transparent, and could easily peel off from the casting plate. The F3 and F4 films were more transparent, flexible, and appeared to be a commercial thin film, such as LDPE. Table 1 shows that the average thickness of fabricated films was around 72 μm, categorized as thin films [37]. F1 was the thickest film with the incorporation of SiO_2_-ZnO nanoparticles. The substitution of CS decreased film thickness due to its amylose content that retracts starch gel during film drying [22]. 

Based on Table 1, the F1 film had the lowest T_280_ value or the highest UV screening due to the UV barrier property of the nanoparticles [38]. Figure 1b shows the transmittance value of bionanocomposite films declined at 365 nm, which is linked to the band-gap energy of the ZnO nanoparticles [34]. Conversely, the F4 film had the highest T_660_ value or the highest transparency. The result was associated with the amorphous structure of CS that transmitted light and created transparent film. The gelatinization process contributed to the change in the semi-crystalline structure to become an amorphous structure, which caused light through the film matrix to become more accesible and appear transparent [39]. Furthermore, the combination of ZnO and SiO_2_ nanoparticles provided better UV screening than those individually incorporated with the carrageenan film [7,8,40]. Then, the addition of CS accelerated the transparency of F2 (%T_660_ = 60.603) to approach the transparency of film-based κ-carrageenan incorporated with halloysite nanotube-Ag (%T_660_ = 73.400) [41]. 

#### 3.1.4. Film Morphology Analysis

**Figure 2:**
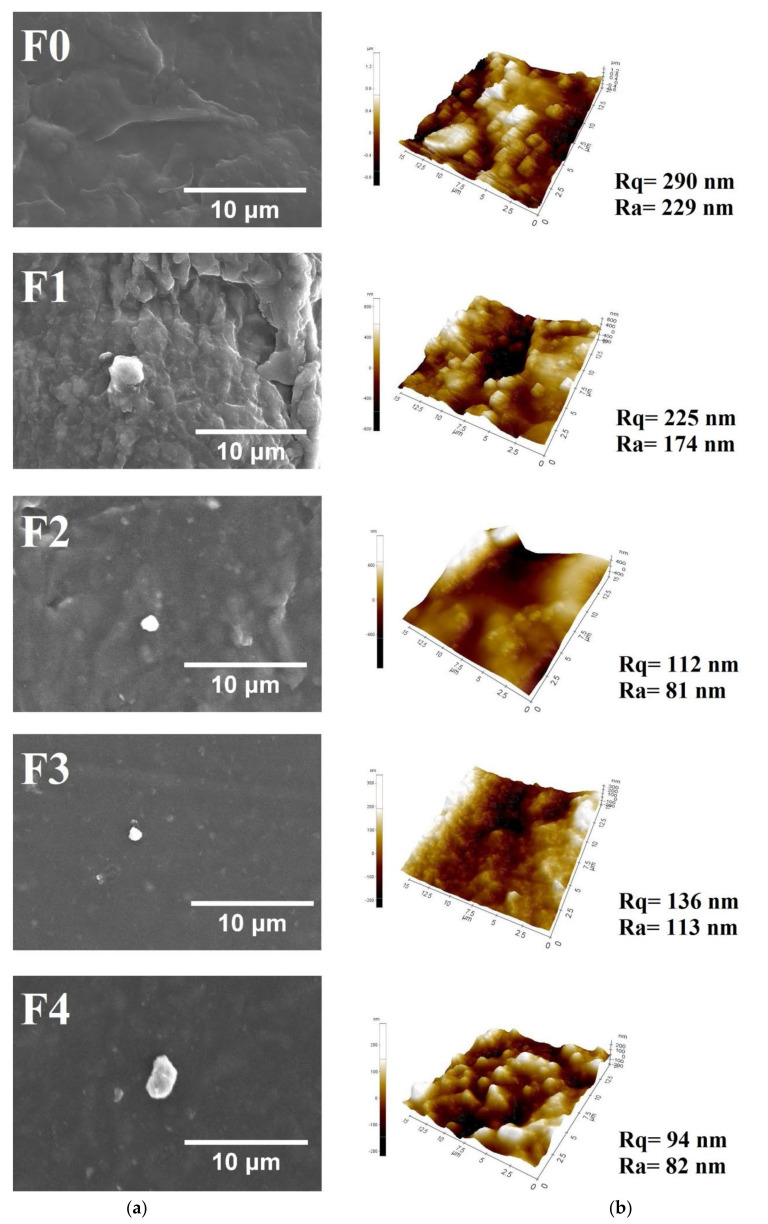
SEM images (**a**) and AFM images (**b**) of the bionanocomposite and control films.

Based on Figure 2a, SEM images of the control film illustrated a rough and heterogeneous surface. Moreover, the incorporation of SiO_2_-ZnO nanoparticles provided a compact film surface, which indicated compatibility between nanoparticles and the polymer matrix [38]. Furthermore, the substitution of CS increased film smoothness due to the amorphous structure of CS. Formation of the amorphous structure improves intermolecular forces and inhibits the mobility of the biopolymer chain and then generates an organized film structure [42]. Figure 2b shows AFM images that depict the film’s smoothness with the substitution of CS, as the SEM result, represented by the lower value of the mean roughness (Ra and Rq). A similar trend was found for chicken skin gelatin-based film [15].

#### 3.1.5. Contact Angle and Critical Surface Tension

Based on Table 2, the water contact angle of fabricated films exceeded 90° and indicated hydrophobicity of the film’s surface [22]. The critical surface tension of the F3 film was the highest and was attributed to the roughness of the film surface, as shown in the AFM result. On the rough surface, adhesiveness was more prominent than cohesiveness, which facilitated liquid for wetting the film surface [20].

#### 3.1.6. Water Vapor Permeability

Based on Table 2, the F1 film had the lowest WVP, associated with the SiO_2_-ZnO nanoparticle performance, which was consistent with FTIR and SEM results. In contrast, the substitution of CS significantly (*p* < 0.05) increased the WVP of fabricated films due to the polar, hydrophilic, and hygroscopic nature of CS, as the FTIR and CST result. The result is comparable to that obtained in a previous study [41,43].

#### 3.1.7. Water Solubility

Table 2 indicates that the water solubility of the F1 and F2 films was significantly (*p* < 0.05) higher than that of other films due to the water-soluble nature of SRiC related to the sulfate ester group, as the FTIR result. The result is similar to that in a previous report by Jancikova et al. [44]. Conversely, increasing the CS proportion significantly (*p* < 0.05) decreased the water solubility of the films due to the amylopectin structure that prevents swelling and decreases water solubility [22]. A similar finding was reported by Loo and Sarbon [15]. 

#### 3.1.8. Mechanical Properties

Based on Table 1, the substitution of CS significantly (*p* < 0.05) decreased the tensile strength of fabricated films. The result is linked to weak interfacial bonding between the polymer matrix and nanoparticles, as the FTIR result, making the film more flexible. Moreover, the tensile strength of fabricated films, except F4, was similar to the Japan Industrial Standard value (minimum 3.92 MPa). However, the elongation of all films was lower than the standard (minimum 70%) [45]. Furthermore, the mechanical properties of developed films were comparable to those in previous studies [12,43].

#### 3.1.9. Thermal Stability

**Figure 3:**
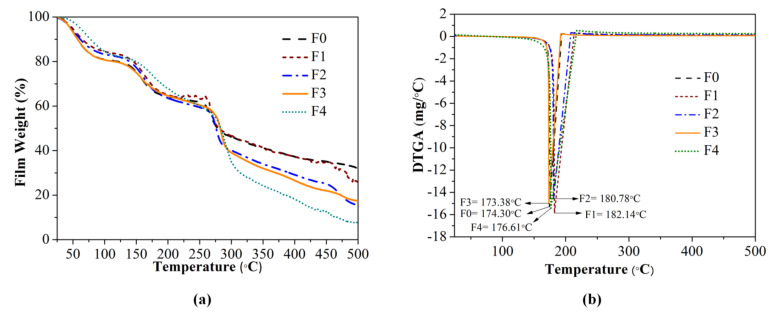
Thermogravimetry analysis (TGA) curve (**a**) and DTGA curve (**b**) of the bionanocomposite and control films. F0: SRiC film (control film), F1: SRiC film with SiO_2_-ZnO nanoparticles, F2: SRiC/CS (1.5 wt%: 0.5 wt%) with SiO_2_-ZnO nanoparticles, F3: SRiC/CS (1.0 wt%: 1.0 wt%) with SiO_2_-ZnO nanoparticles, F4: SRiC/CS (0.5 wt%: 1.5 wt%) with SiO_2_-ZnO nanoparticles.

Figure 3a displays the TGA curve that describes the weight loss of developed films after the final decomposition (at 500 °C). Control film had the lowest residual weight due to the non-ignitable mineral content in SRiC [40]. Figure 3b presents the result of DTGA, which indicates the highest decomposition temperature of the film [23]. The F1 film had the highest thermal stability due to thermally stable ZnO nanoparticles [40]. However, the substitution of CS increased the thermal decomposition of films, associated with the low interaction between the polymer matrix and nanoparticles, as the FTIR result.

#### 3.1.10. Antimicrobial Activity

**Figure 4:**
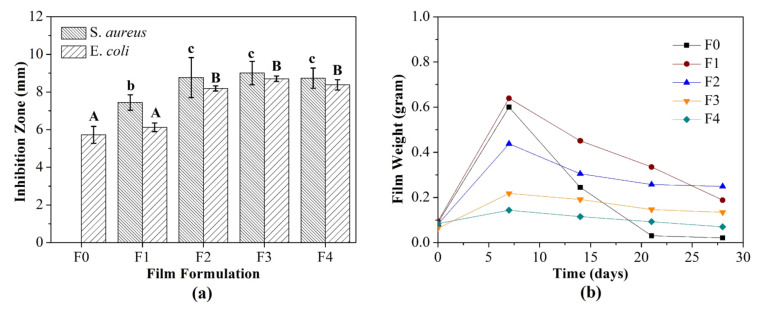
Antimicrobial activity (**a**) and biodegradation rate (**b**) of the bionanocomposite and control films. F0: SRiC film (control film), F1: SRiC film with SiO_2_-ZnO nanoparticles, F2: SRiC/CS (1.5 wt%: 0.5 wt%) with SiO_2_-ZnO nanoparticles, F3: SRiC/CS (1.0 wt%: 1.0 wt%) with SiO_2_-ZnO nanoparticles, F4: SRiC/CS (0.5 wt%: 1.5 wt%) with SiO_2_-ZnO nanoparticles; A,B: uppercase letters indicate significant differences among each film formula inhibited *E. coli*; b,c: lowercase letters indicate significant differences among each film formula inhibited *S. aureus*.

Figure 4a depicts the antimicrobial activity of developed films against *E. coli* and *S. aureus*. Interestingly, the control film presented antimicrobial activity against *E. coli*. A similar result was reported by Padhi et al. [46]. Then, the incorporation of SiO_2_-ZnO nanoparticles improved the antimicrobial activity of films and was more effective against *S. aureus* than *E. coli*. The dense peptidoglycan membrane of *E. coli* inhibited nanoparticle penetration through the membrane cells. A similar trend was reported by Al-Tayyar et al. [11]. Furthermore, the substitution of CS enhanced the antimicrobial activity of films. The increased CS proportion increased the film smoothness, as an AFM result. The smooth surface improved the surface area and increased interfacial interactions between films and bacterial cells. Then, the bacterial cells were more susceptible to disruption by antimicrobial agents that were incorporated into the films. Previous studies have confirmed that the improvement of antimicrobial activity in the film by increasing cassava starch content is related to its water affinity. This phenomenon increased the release of antimicrobial agents, such as nanoparticles [24].

#### 3.1.11. Biodegradability

Figure 4b indicates that the control film was drastically degraded after 28 days, while other films were not fully degraded. The decreased film biodegradation was related to the antimicrobial activity, which decreased the growth rate and infiltration of microorganisms into the film surface [47]. Conversely, the substitution of CS promoted the biodegradation process, attributed to the high WVP level of the films, which was shown by increasing film weight after seven days. This phenomenon supports soil microorganism activity, which reduced the film weight followed by full degradation [25]. Moreover, the metabolic activity of microorganisms also produced hydrolytic enzymes that can digest and degrade the films [48].

### 3.2. Application of Bionanocomposite Films for Minced Chicken Meat Packaging

**Figure 5:**
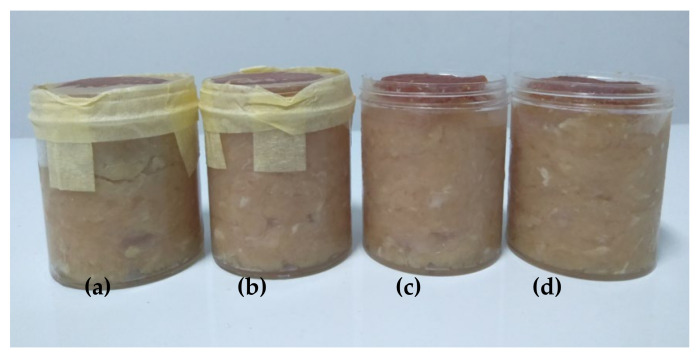
Illustration of the application model of the bionanocomposite film: meat samples packaged with film (**a**,**b**) and without film (**c**,**d**).

The best film formula was determined by the compensatory model using a non-dimensional scaling method [49], as described previously [10]. The results of WVP, water-solubility, mechanical properties, and antimicrobial activity analysis of fabricated films were used to determine the best film formula. Based on the calculation result, F1 and F2 films had the highest scores (0.64) and were selected as the best film formulas. Then, minced chicken packaging was conducted with the unwrapped sample as the control sample (A0) and wrapped sample with selected films, such as F0 film (A1), F1 film (A2), an F2 film (A3), illustrated in Figure 5.

#### 3.2.1. Total Plate Count

TPC evaluates the microbiological quality of meat samples during storage. According to the Indonesian National Standard 3429:2009, the acceptable limit of the TPC value for chicken meat was 6 log CFU/g [50]. Thus, the control sample (A0) was not acceptable after three days of storage, while other samples were still acceptable within six days of storage, as can be seen in Table 3. Additionally, the antimicrobial activity of the F1 and F2 films probably inhibited microbial growth in the samples. A similar finding was reported previously [26,51].

#### 3.2.2. pH

The pH value is attributed to microbial growth and enzymatic reactions during food processing. Generally, the pH of high-quality meat products is around 5.7 to 6.0 [26]. Based on Table 3, A0 had the lowest pH after the 3rd day that was analogous to the TPC result. Other samples had a constant pH and increased after the 9th day. A high pH value is related to alkaline compounds, such as ammonia due to microbial proteolysis. After the 12th day, the pH of all samples ranged from 6.1 to 6.2, which is comparable to previous studies. Application of chitosan-based bionanocomposite film with MMT nanoparticles, ginger essential oil, and rosemary essential oil for minced chicken meat packaging resulted in a pH value of 5.7–7.0 after 15 days of storage [52,53].

#### 3.2.3. Weight Loss

Weight loss is related to the water content and WHC of meat that affect meat quality, consumer acceptance, and meat prices. Table 3 shows the weight loss was significantly (*p* < 0.05) different between sample treatments and storage time. A0 had the highest weight loss, while A1 had the lowest weight loss after 12 days. The result was attributed to the WVP of the film that inhibited water transportation through the film matrix and then reduced the weight loss of the sample. A similar trend was reported by Praseptiangga et al. [54].

#### 3.2.4. Meat Color

Generally, the consumer prefers a bright red meat product, which refers to freshness [26]. Table 3 presents the result of the meat color analysis as the total color difference (∆E), which indicates alternation of meat color during observation. Based on the PCE Instruments Standard, the acceptable limits for ∆E is lower than 4 [55]. Thus, A0 was not acceptable after the 9th day, while other samples were acceptable until 12 days. The result was associated with UV screening of the bionanocomposite film, which restricted meat color oxidation.

#### 3.2.5. Thiobarbituric Acid Analysis

Meat is rich in unsaturated fatty acids that promote lipid oxidation, which causes rancidity and off-flavor. The acceptable limit of the TBA value is 0.5–1.0 mg MDA/kg, which is related to the off-flavor intensity of the sample [56]. Based on Table 3, the TBA of all samples was acceptable until the 3rd day. Nevertheless, the TBA value of A2 and A3 was slightly lower than that of A0, which indicated the UV barrier properties of the bionanocomposite films that prevent lipid oxidation. A similar trend was reported in the previous studies [26,52,53].

#### 3.2.6. Total Volatile Base Nitrogen Analysis

TVBN analysis is associated with meat or fish deterioration by microbial activity and endogenous enzymes, which cause amino acid catabolism and volatile base compounds. The acceptable limit for TVBN is 25 mg N/100 g [32]. Based on Table 3, all samples were acceptable until the 6th day, while A2 was still acceptable until the 9th day. The result was consistent with TPC and pH analysis, which indicated the production of the alkaline compound by microbial metabolism.

## 4. Conclusions

The substitution of CS improved the transparency, smoothness, and biodegradation rate of fabricated films. Incorporation of SiO_2_-ZnO nanoparticles enhanced the UV screening and WVP of the films. CS and SiO_2_-ZnO nanoparticles promoted the antimicrobial activity of the films. The integration properties of SRiC film incorporated with SiO_2_-ZnO nanoparticles (F1) successfully prolonged minced chicken shelf life up to six days based on color and TVBN analysis. Determination of the appropriate storage conditions of the films, such as water vapor adsorption isotherm measurement and the packaging application method, is necessary for future development.

## Figures and Tables

**Table 1:** Thickness, UV screening (T_280_), transparency (T_660_), tensile strength (TS), and elongation at breakage (EAB) of the bionanocomposite and control films.

Film Type	Thickness (μm)	T_280_ (%)	T_660_ (%)	TS (MPa)	EAB (%)
F0	73.375 ± 3.365 ^b^	4.890	24.834	9.131 ± 2.676 ^d^	24.129 ± 9.210 ^a^
F1	83.675 ± 3.430 ^c^	2.238	25.078	7.323 ± 0.595 ^cd^	25.529 ± 2.180 ^a^
F2	69.150 ± 0.777 ^ab^	2.679	27.362	5.194 ± 1.604 ^bc^	27.962 ± 5.768 ^a^
F3	73.425 ± 3.860 ^b^	6.908	60.603	4.192 ± 0.905 ^ab^	19.616 ± 7.494 ^a^
F4	65.200 ± 4.090 ^a^	16.579	68.456	1.907 ± 0.380 ^a^	16.897 ± 9.388 ^a^

Values in the same column followed by different superscript letters are significantly (*p* < 0.05) different.

**Table 2:** The water contact angle (θ_water_), critical surface tension (γ_c_), water vapor permeability (WVP), and water solubility (WS) of bionanocomposite and control films.

Film Type	θ_water_ (°)	γ_c_ (m N/m)	WVP (10^−10^ g/m·Pa·s)	WS (%)
F0	100.967 ± 10.950 ^a^	18.064 ± 1.750 ^ab^	3.072 ± 0.080 ^a^	65.976 ± 2.092 ^c^
F1	94.083 ± 8.228 ^a^	18.635 ± 1.438 ^ab^	2.951 ± 0.067 ^a^	76.341 ± 3.563 ^d^
F2	105.017 ± 5.229 ^a^	18.427 ± 1.092 ^ab^	3.877 ± 0.465 ^b^	81.381 ± 4.340 ^d^
F3	105.250 ± 7.957 ^a^	21.693 ± 2.523 ^b^	4.105 ± 0.378 ^b^	39.210 ± 7.680 ^a^
F4	90.817 ± 5.849 ^a^	16.910 ± 3.680 ^a^	3.847 ± 0.552 ^b^	51.895 ± 7.162 ^b^

Value in the same column followed by different superscript letters are significantly (*p* < 0.05) different.

**Table 3:** Psychochemical and microbiological quality of minced chicken meat packaged with various treatments.

Parameter	Day	A0	A1	A2	A3
TPC (log CFU/g)	0	5.559 ± 0.004 ^aA^	5.559 ± 0.004 ^aA^	5.559 ± 0.004 ^aA^	5.559 ± 0.004 ^aA^
	3	6.145 ± 0.470 ^aA^	5.903 ± 0.269 ^aA^	5.678 ± 0.017 ^aA^	5.698 ± 0.049 ^aAB^
	6	5.980 ± 0.079 ^aA^	6.290 ± 0.059 ^bA^	6.241 ± 0.071 ^bA^	6.191 ± 0.053 ^bAB^
	9	6.069 ± 0.381 ^aA^	6.029 ± 0.106 ^aA^	6.295 ± 0.078 ^aA^	7.025 ± 0.087 ^bAB^
	12	6.444 ± 0.878 ^aA^	6.307 ± 0.336 ^aA^	7.734 ± 0.157 ^aA^	7.513 ± 0.826 ^aB^
pH	0	5.987 ± 0.025 ^aAB^	5.987 ± 0.025 ^aA^	5.987 ± 0.025 ^aA^	5.987 ± 0.025 ^aA^
	3	5.791 ± 0.460 ^aA^	6.047 ± 0.011 ^aB^	6.001 ± 0.021 ^aA^	6.018 ± 0.008 ^aB^
	6	6.062± 0.015 ^aAB^	6.080 ± 0.008 ^aBC^	6.078 ± 0.008 ^aB^	6.071 ± 0.017 ^aC^
	9	6.107 ± 0.003 ^aAB^	6.301 ± 0.054 ^dD^	6.237 ± 0.018 ^cC^	6.182 ± 0.003 ^bE^
	12	6.163 ± 0.011 ^abB^	6.127 ± 0.037 ^aC^	6.210 ± 0.084 ^bC^	6.123 ± 0.005 ^aD^
WHC (%)	0	108.611 ± 2.678 ^aD^	108.611 ± 2.678 ^aC^	108.611 ± 2.678 ^aD^	108.611 ± 2.678 ^aC^
	3	87.783 ± 2.163 ^aB^	96.398 ± 1.282 ^bB^	98.405 ± 0.806 ^bC^	86.753 ± 1.941 ^aAB^
	6	90.153 ± 1.165 ^bBC^	89.278 ± 1.772 ^bB^	83.636 ± 1.616 ^aB^	88.190 ± 2.804 ^bAB^
	9	83.312 ± 3.244 ^bA^	79.629 ± 2.423 ^bA^	72.138 ± 1.995 ^aA^	84.653 ± 4.818 ^bA^
	12	92.502 ± 2.622 ^aC^	90.508 ± 9.359 ^aB^	86.200 ± 1.004 ^aB^	91.291 ± 2.395 ^aB^
WL (%)	0	0 ^aA^	0 ^aA^	0 ^aA^	0 ^aA^
	3	9.148 ± 2.504 ^bB^	3.545 ± 0.599 ^aB^	4.269 ± 0.310 ^aB^	3.880 ± 0.516 ^aB^
	6	13.405 ± 0.639 ^cC^	4.668 ± 0.193 ^aC^	5.517 ± 0.287 ^bC^	5.994 ± 0.625 ^bC^
	9	19.044 ± 0.669 ^cD^	5.431 ± 0.227 ^aD^	6.593 ± 0.423 ^bD^	7.402 ± 0.903 ^bD^
	12	23.498 ± 1.111 ^dE^	6.810 ± 0.164 ^aE^	8.228 ± 0.246 ^bE^	10.172 ± 1.014 ^cE^
ΔE	0	-	-	-	-
	3	3.412 ± 0.137 ^bA^	2.526 ± 0.276 ^aB^	3.300 ± 0.292 ^bB^	2.795 ± 0.389 ^aA^
	6	3.005 ± 0.724 ^aA^	3.886 ± 0.612 ^aC^	3.620 ± 0.109 ^aB^	2.976 ± 0.715 ^aA^
	9	5.081 ± 0.066 ^bB^	3.061 ± 0.681 ^aB^	2.310 ± 0.209 ^aA^	3.033 ± 1.790 ^aA^
	12	8.987 ± 0.011 ^dC^	1.265 ± 0.277 ^aA^	2.146 ± 0.978 ^bA^	3.717 ± 0.336 ^cA^
TBA (mg MDA/kg sample)	0	0.293 ± 0.130 ^aA^	0.293 ± 0.130 ^aA^	0.293 ± 0.130 ^aA^	0.293 ± 0.130 ^aA^
	3	0.650 ± 0.061 ^aB^	0.656 ± 0.060 ^aBC^	0.561 ± 0.115 ^aB^	0.578 ± 0.104 ^aB^
	6	0.865 ± 0.138 ^bBC^	1.059 ± 0.029 ^cD^	1.014 ± 0.142 ^bcC^	0.703 ± 0.060 ^aB^
	9	0.984 ± 0.240 ^bC^	0.802 ± 0.205 ^abC^	0.680 ± 0.032 ^aB^	0.676 ± 0.004 ^aB^
	12	0.712 ± 0.213 ^aB^	0.566 ± 0.014 ^aB^	0.719 ± 0.064 ^aB^	1.131 ± 0.236 ^bC^
TVBN (mg N/100 g)	0	5.423 ± 0.063 ^aA^	5.538 ± 0.109 ^aA^	5.423 ± 0.063 ^aA^	5.538 ± 0.109 ^aA^
	3	25.248 ± 1.300 ^Cb^	19.570 ± 0.008 ^aB^	21.445 ± 0.073 ^bB^	24.478 ± 0.105 ^cB^
	6	23.848 ± 1.074 ^cB^	21.018 ± 1.518 ^aB^	21.965 ± 0.303 ^abB^	23.273 ± 0.587 ^bcC^
	9	35.878 ± 2.065 ^cC^	29.663 ± 0.120 ^bC^	23.478 ± 0.428 ^aC^	31.035 ± 0.450 ^bD^
	12	67.875 ± 1.738 ^cD^	54.993 ± 1.994 ^bD^	45.635 ± 1.658 ^aD^	45.458 ± 0.587 ^aE^

Mean values in the same column with different lowercase letters (a–d) indicate significant differences among formulations within each parameter. Mean values in the same line with different uppercase letters (A–E) indicate significant differences among days within each parameter. A0: control meat sample (without film/ uncovered), A1: sample is packaged with F0 film, A2: sample is packaged with F1 film, A3: sample is packaged with F2 film, TPC: total plate count, WHC: water holding capacity, WL: weight loss, ΔE: total color difference, TBA: total barbituric acid, and TVBN: total volatile base nitrogen).
